# The meaning of “total pain” in the context of living and dying with dementia

**DOI:** 10.3389/fsoc.2024.1412749

**Published:** 2025-04-09

**Authors:** Sarah Elizabeth Field-Richards, Louise Bramley, Jemima Collins, Alison Cowley, Rowan Harwood

**Affiliations:** ^1^School of Health Sciences, University of Nottingham, Nottingham, United Kingdom; ^2^Nottingham University Hospitals NHS Trust, Nottingham, United Kingdom; ^3^School of Medicine, University of Nottingham, Nottingham, United Kingdom

**Keywords:** dementia, pain, total pain, palliative care, end of life care, person-centred care, personhood, social construction

## Abstract

**Background:**

Globally, there are 55 million people living with dementia (PLWD). PLWD have an uncertain prognosis. Most are approaching the end of life but are not overtly or immediately dying. Contemporary approaches to dementia care therefore promote the need to live and die well with dementia. Pain is highly prevalent but difficult to manage in PLWD. Originating in palliative care, “total pain” conceives of pain holistically, incorporating biological, psychological, social and spiritual elements. Pain management in dementia care tends to be pharmacologically focused. Total pain therefore offers an alternative approach—one consistent with person-centred philosophy underpinning contemporary dementia care. Due to important differences, concepts cannot simply be extrapolated from cancer-related to dementia-related palliative care however. Dementia-specific approaches are needed and require exploration.

**Description and objective of the analysis:**

The objective of this paper is to explore the meaning of total pain in the context of living and dying with dementia, and its utility and implications for person-centred dementia care. Using a palliative care framework and existing literature, we critically consider the bio-psycho-socio-spiritual impact of dementia, to explore how total pain might manifest and be experienced in this context.

**Findings and interpretation:**

We highlight the complexity, nuance and socially contingent nature of the impact of living and dying with dementia. We challenge binary understandings of “continuity or loss” (e.g., of identity, relationships), and totalising “loss” discourses, demonstrating that more subtle, varied and hopeful outcomes are possible. The way that the impact of dementia is articulated and understood has implications for the experience and management of total pain. The deficit-orientation of “total *pain*” paradoxically risks its perpetuation. A balanced understanding of dementia’s impact (acknowledging *both* continuity and loss, alternatives and socially constructed aspects) better reflects the realities of dementia and creates new possibilities for supportive care practices to improve pain management and quality of life.

**Conclusion and implications for practice:**

Applied to dementia care, “total pain” should be located within a critical context, emphasising complexity, contingency and nuance. The holistic focus of “*total* pain” should be extended to incorporate balanced consideration of “painful” and “functional” experience. We introduce a balanced model of total pain incorporating a dual focus on “pain” and “personhood” within a critical context, to facilitate translation to practice. There is a need to develop evidence-based supportive interventions in each domain of total pain, to support a balanced approach to total pain management in dementia care.

## 1 Introduction

Biomedically, “dementia” encompasses a range of progressive and irreversible neurodegenerative disorders (McKhann et al., 2011). It causes a decline in cognitive function including thinking, memory, comprehension, language, judgement, and changes in emotional control, mood, motivation and behavior (World Health Organization, 2021a). According to the World Health Organization (2021a), there are 55 million people living with dementia (PLWD) globally, which is a leading cause of disability, dependency and mortality. This number is expected to rise to 78 million by 2030 and to 139 million by 2050 (World Health Organization, 2021b). There is currently no known cure for dementia.

PLWD face an uncertain prognosis. Physical and mental co-morbidity is almost universal and functional decline can be rapid when acute illness or injury occur (Jackson et al., 2017). Modern geriatric medicine uses the term “frailty” to describe vulnerability to crises and functional decline, as seen in PLWD. Problems accompanying dementia are often functional – immobility, falls, incontinence and challenging behaviors, leading to dependency (Goldberg et al., 2021). Dependency refers to the need for help from others, often spouses who are themselves frail, and adult children with competing domestic and work commitments.

Most PLWD are approaching the end of life but are not overtly or immediately dying. Contemporary approaches to dementia care therefore promote the need to live well, as well as die well, with dementia (Jackson et al., 2017). With the prevalence of dementia increasing and in the absence of a cure, dying with or from dementia will become increasingly common (WHO, 2021a, van der Steen et al., 2014). Living and dying well with dementia therefore constitutes a growing contemporary challenge and research priority (Eisenmann et al., 2020; NHS England, n.d.).

In this paper we explore the meaning of “total pain” in the context of living and dying with dementia, and its utility and implications for person-centred dementia care. In the remainder of this introduction, we introduce the key concepts underpinning our analysis (person-centred and palliative care, pain and total pain), and locate them in the context of contemporary dementia care. An overview of the paper and structure of the analysis is then presented.

### 1.1 Contemporary approaches to dementia care

#### 1.1.1 Person-centred care and personhood

Person-centred care, associated with social psychologist Tom Kitwood, is the dominant philosophy underpinning contemporary dementia care. Kitwood (1990, 1997) moves away from the biomedical “standard paradigm” towards a holistic, biopsychosocial, dialectical model, where respect for and preservation of “personhood” is central. Following Buron (2008:324), personhood refers to characteristics “associated with being a person”. They suggest that one’s personhood status is not fixed but is mutable according to physical and mental health, and age, for example. Considerations of personhood are particularly relevant at the beginning and end of life—to ask when a person begins and ceases to be a person is a question of personhood.

Buron (2008) identifies biologic, individual and sociologic levels of personhood. Biologic personhood relates to a person’s ability to feel pain and pleasure (sentience). At the individual level, characteristics such as personality, past roles, values, spirituality, self-awareness, psychological continuity (the ability to connect past and present), communication, moral agency and cognitive functioning, identify personhood. Personhood at an individual level may be threatened as dementia progresses, and confined to the biologic level. Sociologic personhood is socially defined, by society’s perceptions and treatment of PLWD. It is assigned in accordance with, for example, social relationships and responsibilities, culture and group memberships. It is frequently the first aspect to be affected by dementia, as individuals behave differently towards PLWD following awareness of cognitive changes, leading to PLWD becoming socially isolated, risking loss of personhood at this level.

Supporting personhood is the central goal of person-centred dementia care and involves treating individuals with dignity, respect, and in a manner supportive of their sense of self (Hennelly et al., 2018). Valuing the individual, addressing physical and mental health, acknowledging biography, preferences, empathy and using relationships to provide comfort, attachment and occupation, are important. Personhood can be challenged however through psychosocial processes, including decline in cognitive abilities, independence, and (often unintentionally) through the way PLWD are spoken to and interacted with (e.g., infantilisation, objectification, exclusion, dishonesty).

#### 1.1.2 A palliative approach

As a life-limiting disease without a cure, the palliative care approach traditionally associated with cancer diagnoses is receiving increased attention in dementia care (van der Steen et al., 2014). Palliative care aims to improve quality of life for patients and families, supporting patients to live as actively as possible, using a holistic approach to the prevention and relief of suffering associated with life-threatening illness (World Health Organization, 2020). Owing to co-morbidity, behavioral symptoms, communication issues and the particularly intense and often lengthy nature of the family caregiver role involving proxy decision-making, palliative requirements for PLWD are unique and dementia-specific approaches are required (*ibid.*, Eisenmann et al., 2020). Cancer and dementia disease progression trajectories also differ significantly (Harris, 2007). The progression of dementia is not linear, and prognostication and terminal phase identification are difficult (Eisenmann et al., 2020; Harris, 2007). End of life care should therefore form an integral part of “routine” dementia care (*ibid.*).

**Figure 1:**
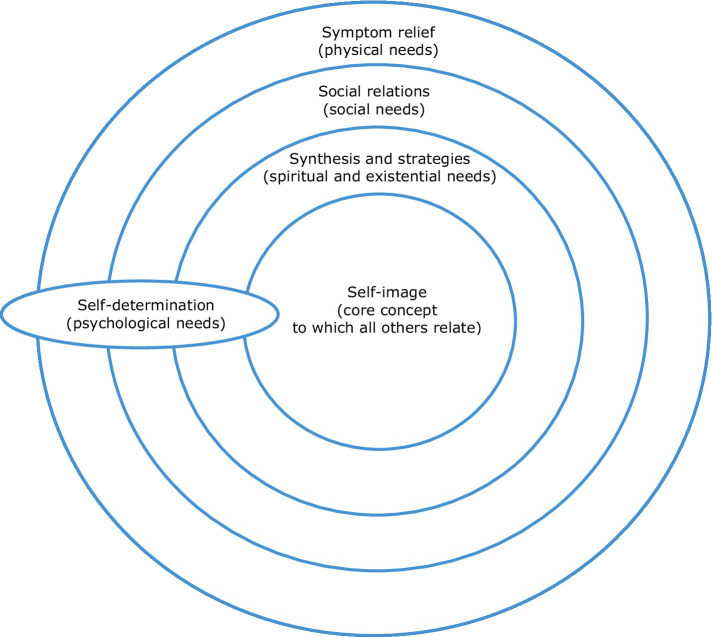
The 6S-model for person-centred palliative care. Adapted from Österlind and Henoch (2021:2).

Österlind and Henoch (2021:2) propose a theoretical model to support the provision of person-centred palliative care. As a person-centred model, personhood (“seeing the patient as a person…a thinking, feeling, interpreting, social and creative being”) is acknowledged as central. The “6S-model” (Figure 1) comprises 6 inter-related concepts, describing an individual’s holistic dimensions and needs: Self-image (the core concept, to which other dimensions relate), Synthesis and Strategies (spiritual and existential needs), Social relations (social needs), Symptom relief (physical needs), and Self-determination (psychological needs and an integrative concept). This model will be expanded upon in succeeding sections of the paper.

### 1.2 Pain and dementia

Biomedically, pain is defined as “An unpleasant sensory and emotional experience associated with, or resembling that associated with, actual or potential tissue damage” (International Association for the Study of Pain, 2020: para 3). Pain is highly prevalent in PLWD and is associated with multiple types (e.g., nociceptive, neuropathic, orofacial) and sources (e.g., gastrointestinal, musculoskeletal, decubitus ulcers, genitourinary infections, cardiac conditions) (Achterberg et al., 2020). Assessment of pain is difficult however, due to poor verbal expression and the inability to use abstract concepts and recall temporal changes, that accompany dementia (Achterberg et al., 2020; Sampson et al., 2015; Rodger et al., 2015). Indeed it is well noted that whilst individuals experiencing more severe cognitive impairment are more likely to exhibit pain according to observational measures, they are less likely to communicate experiencing pain verbally (Resnick et al., 2022). Unmanaged pain contributes to functional impairment (with the potential to further exacerbate pain) and negatively influences quality of life more broadly (*ibid.*). Significantly, pain may underlie “challenging” behaviors (e.g., aggression, calling out, agitation, withdrawal), which are generally interpreted as indicating (communicating or expressing) unmet need (Sampson et al., 2015; Beaver et al., 2020).

Despite dementia care promoting a person-centred approach, pain management in PLWD tends to be pharmacologically focused. PLWD are exquisitely sensitive to the adverse effects of many drugs however, especially opioid analgesia, often resulting in delirium, falls, constipation and poor appetite (Rodger et al., 2015; Kanagaratnam et al., 2016). Most other analgesic drug classes (e.g., nonsteroidal anti-inflammatory drugs, nefopam) are equally contraindicated in an older, frail population typical of PLWD. Moreover, their effectiveness is at best uncertain. Whilst it is often suggested that analgesics are under-prescribed in PLWD (Rodger et al., 2015), the balance of treatment benefit and burden is usually unclear or adverse. Similar concerns have arisen regarding use of anti-psychotic and other psychotropic drugs for “challenging” behaviors (Banerjee, 2009). Pain management, and the related management of “challenging behaviors”, therefore remains problematic in PLWD and requires further consideration.

### 1.3 The concept of total pain

Originating from the work of Cicely Saunders in palliative care, “total pain” moves beyond the biomedical model, towards a more holistic conception incorporating physical, psychological, social and spiritual elements, including consideration of family caregivers (Clark, 1999; Mehta and Chan, 2008; Saunders et al., 1995). According to this model, appreciation of the socio-cultural and biographical context in which pain is located and experienced is essential to understanding and addressing it, and to relieving suffering and distress (Clark, 1999; Saunders et al., 1995).

**Figure 2:**
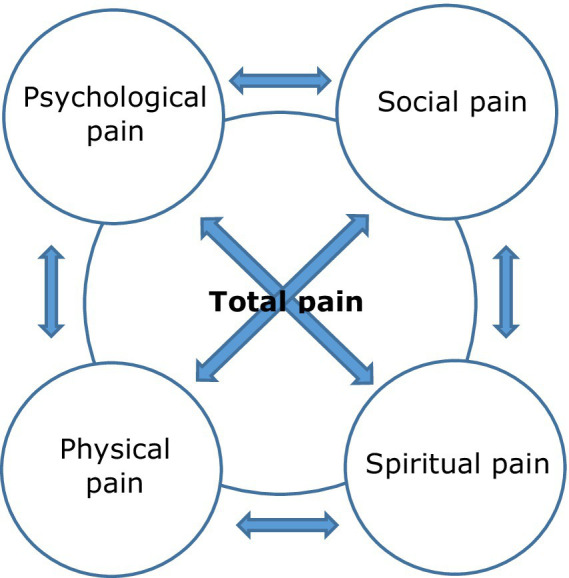
A depiction of Saunders et al.’s (1995) concept of total pain.

The components of total pain interact and are interrelated. Distress experienced in relation to one domain influences and is influenced by others (Saunders et al., 1995; Goebel et al., 2009). Physical pain therefore influences and is influenced by other domains in a bidirectional fashion and effective pain management is unlikely unless all aspects are addressed (Saunders et al., 1995). Saunders suggests that attending to total pain reduces the need for analgesics and sedatives (Clark, 1999). Figure 2 provides a depiction of Saunders et al (1995)’s total pain model.

### 1.4 The complementarity of total pain, palliative and person-centred care

The concept of total pain resonates strongly with the person-centred philosophy underpinning contemporary dementia care. In both cases, human need is holistically conceived of, with unmet need foregrounded as a source of distress and suffering, and the stimulus and necessary response biopsychosocial. In this context, “challenging” behavior in PLWD can be interpreted as a manifestation and performative aspect of total pain – reflective of unmet physical, psychological, social and spiritual need. Recognition of the impact of (and on) family caregivers is also a shared priority.

**Figure 3:**
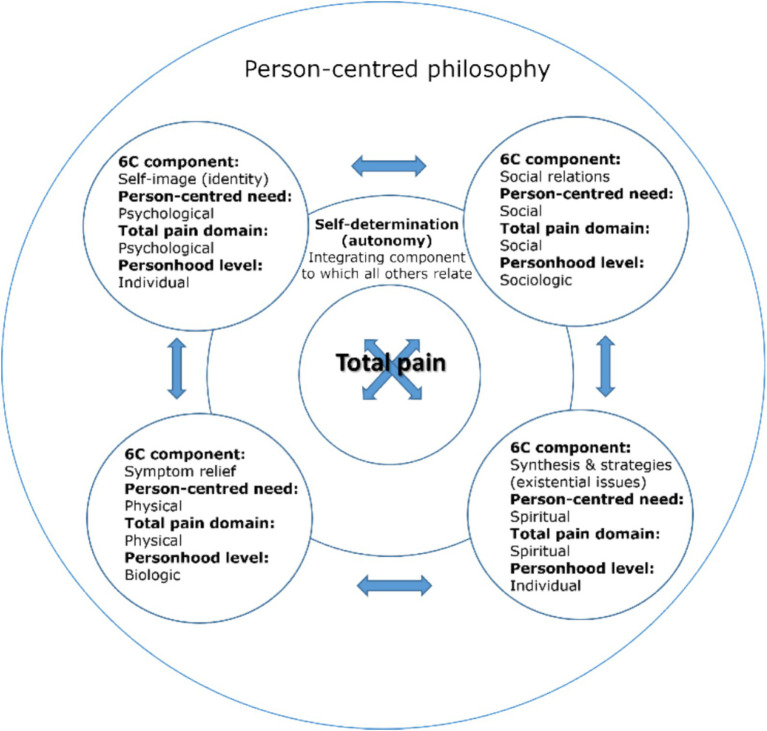
A combined model of total pain (Figure 2), person-centred palliative care (Figure 1) and personhood (Buron, 2008), supported by person-centred philosophy (Kitwood, 1990, 1997).

The components of total pain can be mapped onto and amalgamated with those of Österlind and Henoch’s (2021) 6S model of person-centred palliative care.^1^ Further, this combined model is compatible with the person-centred philosophy underpinning dementia care, including Buron’s (2008) aspects of personhood, attentive to biopsychosocial need. Figure 3 depicts this amalgamation of total pain, person-centred palliative care and personhood, supported by person-centred philosophy.

### 1.5 Total pain in the context of a person-centred palliative approach to dementia care

Given (a) the complementarity of total pain, person-centred and palliative care, (b) the impetus for a person-centred palliative approach to dementia care, combined with (c) the need to address shortcomings of biomedical pain management in this population, we suggest that the concept of total pain may hold utility for supporting a person-centred palliative approach to dementia care—in particular, a person-centred framework for pain management.

In the same way that cancer and dementia disease progression trajectories differ, the palliative requirements of PLWD are also unique. Co-morbidity, behavioral symptoms, communication issues, and the particular nature of family caregiver roles (involving proxy decision-making, high intensity and often long duration), require dementia-specific approaches (van der Steen et al., 2014). As a corollary of such “special issues” of palliative dementia care, insights from cancer-related palliative care “cannot simply be transferred” to this population (Eisenmann et al., 2020:2). The facets of total pain in dementia care might therefore be considered to manifest uniquely. The meaning of total pain in context of living and dying with dementia therefore requires exploration, in order to consider potential challenges, opportunities, and assess its ability to contribute usefully to and improve the quality of dementia care.

### 1.6 Overview and structure of the analysis

In this paper, we explore the meaning of total pain in the context of living and dying with dementia and its implications for the provision of person-centred dementia care. The structure of our analysis is guided by the combined model of total pain and person-centred palliative care, depicted in Figure 3. We focus predominantly on the psychological, social and spiritual domains, as biomedically defined physical pain (relating to symptom relief in the 6S model) is the normative “standard paradigm” understanding of pain in dementia and had been introduced in 2.1. Although we do not therefore expand on this component directly, a biomedical perspective is considered in the context of arguments presented in relation to other components throughout the paper. For each remaining aspect of the 6S model, we describe its nature then, drawing on existing dementia literature, we critically consider how the aspect of total pain to which it is aligned might manifest and be experienced in the context of living and dying with dementia. Use of the 6S model to structure our analysis ensures its relevance to person-centred palliative care, as the advocated approach to supporting living and dying with dementia. Mapping facets of total pain onto the 6S model allows us to consider each aspect of total pain in turn, as it relates to person-centred palliative care, and ensuring a comprehensive exploration. Drawing on existing dementia literature to consider how aspects of total pain manifest in this context ensures relevance to person-centred palliative *dementia* care and allows the identification of relevant implications for practice. This approach therefore addresses both “the meaning of total pain” (critically considering how total pain manifests and its implications) and “in the context of living and dying with dementia” (through use of the 6S model and dementia specific literature).

In accordance with the components of the combined model of total pain and person-centred palliative care (Figure 3), in the subsections of 2.0 below, section 2.1 focuses on self-image (identity) and the psychological domain of total pain, section 2.2 on social relations and the social domain of total pain, section 2.3 on synthesis and strategies (existential issues) and the spiritual domain of total pain, and section 2.4 focuses on self-determination (autonomy) and the integration of total pain domains.

## 2 The meaning of total pain in the context of living and dying with dementia

### 2.1 Self-image (identity) and the psychological domain of total pain

This section considers the meaning of total pain in the context of living and dying with dementia, in relation to psychological pain and the self-image (identity) component of the 6S model for person-centred palliative care. Within the model, self-image is considered to be synonymous with identity, and related to concepts of self and personality. Self-image refers to a person’s needs, beliefs and characteristics that should inform care, to preserve identity. Hennelly et al. (2018) consider sense of self, self-identity and selfhood to be core elements of personhood, and self-image (identity) resonates with the individual level of personhood identified by Buron (2008).

The 6S model depicts self-image as its “core concept”, to which other components relate, and does not associate it with a specific holistic need (see Figure 1). In this analysis however, self-image (identity) is interpreted to correspond with psychological needs, and the psychological domain of the total pain model (depicted in Figure 3). Whilst it is acknowledged that self-image relates to other domains, the self is undoubtedly a psychological construct and one of particular relevance in the context of living and dying with dementia, therefore warranting discrete discussion in this exploration. In this analysis, it is self-determination (explored in 2.3) that forms the integrative concept, which also serves a uniting function in the original model (see Figure 1).

Aligning with the psychological domain of total pain, any challenges to self-image (identity) presented by dementia could be interpreted as potential sources of psychological pain (as psychologically painful). That is, the impact of dementia on self-image constitutes a potential source of psychological pain. This section draws on existing literature relating to self-image and dementia, to explore this psychological aspect of the experience of total pain in the context of living and dying with dementia, and its implications for person-centred dementia care.

#### 2.1.1 Loss, continuity, and the self in flux

Literature suggests that dementia has a profound effect on the self. More specifically, dementia-related challenges and changes to the self are frequently interpreted as manifesting as identity loss, or loss of self (see, e.g., Norberg, 2019, Harding and Palfrey, 1997, Scott, 2022). Associated with individualistic, cognitive notions of the self (Fuchs, 2020), Hampson and Morris (2016:25) for example, note understandings of dementia centred around “decay, decline and deficiency”, in which an individual becomes a “non-person” as a consequence of the erosion of the self, associated with a decline in cognitive functioning. In accordance with this view, awareness of the self “slipping away”, might be interpreted as psychologically painful (to both PLWD and their carers) within the total pain model.

Other literature challenges such determinism however, presenting a more nuanced picture. In exploring the impact of dementia on sense of self, Scott (2022:508, 513) found that dementia negatively impacted participants’ self-esteem, changed self-perception and self-concept, with individuals reporting feeling like “a different person”, accompanied by feelings of powerlessness, anxiety, depression and loneliness. Strategies were also identified however, that demonstrated the management of self-presentation to reduce impact on selfhood. These included active consideration of whether to disclose dementia diagnosis (disclosure practices), concealment of symptoms (to prevent stigma, appearing “stupid” or “rude”, or to avoid others feeling awkward or embarrassed), negotiation of boundaries to avoid situations which threatened self-worth, and concentration on preserved rather than “lost” capabilities. In this sense, although dementia could impact “negatively” on the self, participants described adaptations to sense of selfhood and strategies to lessen impact. Similarly, in their synthesis of qualitative evidence relating to personhood from the perspectives of PLWD, Hennelly et al. (2021) identify that individuals adopted strategies to actively maintain and project their sense of self, by using pronouns (I, me), narration of self through life-story articulation, and image management to preserve identity and self assertion, for example. In these instances, selfhood could be considered to be actively maintained (or steps taken towards this), in addition to aspects considered to be lost, suggesting that whilst dementia can present challenges to identity, this does not necessarily equate with a simplistic notion of “loss”.

In their review of lived experiences of wellbeing in PLWD, Clarke et al. (2020:9) report the potential to experience a positive sense of self-worth, self and identity, and “transcend” the impact of dementia through methods that maintain identity. Similarly, Wolverson et al. (2016:695) report the theme of “still being me”, describing the preservation of sense of self, identity and the importance of maintaining this, as a concern for PLWD. Participants in Phinney’s (2011:258) study spoke of being “the same person they had always been”, with the most self-defining, central part of them protected from dementia, despite sometimes significant change to their lives.

This account suggests that a binary understanding emphasising loss on one hand, or continuity on the other, is inadequate for understanding the impact of dementia on the self. Rather, PLWD can experience *both* aspects of continuity and loss, with neither absolute or total. Phinney (2011) adds that in considering simplistically whether the self is “lost” or “not lost” in dementia, changes, shifts and flux associated with the self are also overlooked, further limiting nuance of understanding.

#### 2.1.2 The socially contingent, negotiated self

Hampson and Morris (2016) highlight perspectives that similarly challenge dualistic understanding (including that the self may be fragmented, concealed and changed), together with social constructionist insights. Rather than considering whether the self is lost or maintained, predicated on biomedical and individualistic mechanisms, social constructionist understandings shift the focus towards the role of social processes in understanding the impact of dementia on the self. The self is seen in social rather than cognitive terms (Hampson and Morris, 2016) and can therefore be understood as socially contingent. Sabat and Harré (1992) for example, draw on the social construction of dementia and the discursive production and maintenance of the self, to argue that the self is not “lost” as a consequence of the dementia disease process but can be denied and lost *indirectly*, as a consequence of the perceptions of and treatment by others. As construction is dependent upon recognition, response, and confirmation by others, a particular version of the self can come into being in communication with one person but not with another, if their cooperation in the construction is absent. In turn, the version of the self that is socially constructed then impacts an individual’s social positioning in a given situation. Here, the self in PLWD might therefore be considered negotiated, as well as contingent.

#### 2.1.3 Positioning and a self-fulfilling prophecy

Similar to labelling theory, according to “positioning theory”, Sabat and Harré (1992) suggest that once an individual with dementia has been positioned in a certain way (commonly as “confused” or “helpless”), subsequent behavior is likely to be interpreted in a way that conforms to and accords with the storyline of that positioning. This undermines the self attempting to be presented by PLWD and leads to a self-fulfilling prophecy. That is, positioning (or labelling) an individual as confused defines the parameters of behavioral interpretations available. Alternative interpretations are rejected, speech and behavior are interpreted accordingly, seemingly “confirming” an individual’s “confusion”. Sabat and Harré (1992) therefore suggest that the greatest threat to the self in PLWD comes not from the disease itself but from the behavior of others in their reactions and treatment. Further, essentially reflecting the internalisation of a given social label, Scott’s (2022:514) research suggests that the views of others can become reflected back onto PLWD, who come to view “themselves in accordance with how others see them”, with the conceptualisation of dementia again mediating its psychological impact. This could be seen to extend and compound the positioning prophecy and impact on the self.

#### 2.1.4 Implications of the psychological aspect of total pain for person-centred dementia care

How we understand the impact of dementia on the self has implications for not only the way PLWD experience psychological pain itself, but also for its management and the delivery of person-centred care (in turn further influencing the experience of psychological pain).

Hampson and Morris (2016) argue that it is important to consider the way in which the self is viewed in dementia, as this can have a significant impact on how the caregiver role is positioned in relation to supporting the self, and levels of disability and wellbeing. They suggest that when viewed as “lost”, the ethical requirement to provide care is diminished and provision can be reduced to meeting physical needs. Viewing the self as fragmented or concealed however, permits care beyond physical need, promoting meaning and wellbeing more broadly, consistent with person-centred care. For Buron (2008), this equates with the presence or absence of individual personhood – without which, care is directed to the physical level. Similarly, Phinney (2011:267) and George (2010:587) suggest that developing understanding of ways in which PLWD can be supported requires seeing “beyond the stark contrast” presented by the “continuity versus loss” proposition, and a more humane approach might be facilitated through encouraging a social view of dementia as a *change* in self. This is not to deny that dementia can affect identity in ways that can be challenging and distressing, but “a more sensitive understanding” illuminates that “identity is never completely lost until death”.

The social constructionist perspective highlights the influence that healthcare professionals can have on psychological pain outcomes, and indicates ways in which this aspect of total pain might be addressed. The way that caregivers interpret and understand the impact of dementia on the self influences how they communicate with and care for PLWD. This in turn contributes to possible constructions of the “self”, possibilities for care, and potentiates (or mediates) psychological pain. Managing the psychological aspect of total pain in PLWD therefore requires being attentive and attending to this dynamic. This might be achieved by adopting a nuanced, individualised, socially contingent understanding of the impact of dementia on the self, signalling acknowledgement that a binary understanding is inadequate for accommodating the complex and disparate nature of experience, and a deterministic “loss” understanding constrains possibilities for the self through the actions of a self-fulfilling prophecy. Such an appreciation introduces a more abling orientation that prevents or lessens (socially constructed) psychological pain associated with an “inevitable loss” of the self, and promotes possibility and support of selfhood.

### 2.2 Social relations and the social domain of total pain

This section considers the meaning of total pain in the context of living and dying with dementia, in relation to social pain and the social relations component of the 6S model. Österlind and Henoch (2021) suggest that relationships provide the opportunity to share health-related changes and losses, and acknowledging the close relationship between the social and psychological, membership of a social community can assist in the maintenance of self-image/identity. Similarly, the importance of social relationships in supporting psychological needs are emphasised in definitions of personhood. McCormack et al. (2012:286) for example, describe personhood as “a sense of self-identity maintained by relationships”, involving effective social interaction. Personhood is thus “socially constructed and maintained” (*ibid.*), and these social and relational aspects of personhood correspond with the sociologic level of Buron’s (2008) model.

The social relations component of the 6S model corresponds with an individual’s social needs and the social domain of total pain (Figure 3). The impact of dementia on social relations can therefore be considered a potential source of social pain (as socially painful). This analysis draws on existing literature relating to social relations and dementia, to explore the social aspect of the experience of total pain in the context of living and dying with dementia, and implications for person-centred care.

#### 2.2.1 Social death and stigma

Research by Scott (2022:509) suggests that dementia impacts social identity and roles, such as those associated with caring (no longer caring for children in the family, for example). Changes to status as “the one in charge” and relied upon by others, central to gendered and familial role identities, can in turn threaten sense of self. Participants also described social withdrawal due to social anxiety, and consequent loneliness.

The concept of “social death” has long been prevalent in the dementia literature. Here, following cumulative “losses” (e.g., of identity, social relationships, the ability to participate in daily activities), individuals may be “discounted” and regarded as “as good as dead” in social terms—and treated as such (Sweeting and Gilhooly, 1997:99; Borgstrom, 2017:6). Dementia is also noted to be highly stigmatised, including by healthcare professionals, with significant negative implications for quality care and the health and wellbeing of PLWD and their carers (Kim et al., 2019; Herrmann et al., 2018; Aboseif and Woo, 2020). Consequently, the topic of stigma is receiving increasing attention in the dementia context (Aboseif and Woo, 2020).

Social death and stigma can be observed to be mutually constitutive and reinforcing, with stigma acting as both a precursor to and sequela of social death. “Stigma” refers to an undesirable attribute or “differentness”, that demarcates an individual from others in a social category (the “normals”), with a deeply discrediting effect (Goffman, 1968:15). Affected individuals are considered tainted, socially disqualified and discounted as “not quite human” (*ibid.*). Link and Phelan (2001) define stigma as the co-occurrence of *labelling* (of difference), *negative stereotyping* (linking labelled differences to undesirable characteristics via dominant cultural beliefs), *separation* (categorising labels to establish separation between “us” and “them”), *status loss and discrimination* (rejection, exclusion, leading to unequal outcomes), occurring in a *power situation* (stigmatisation is dependent on socioeconomic, political power to enact the components). Kim et al. (2019:2) add that stigma can extend to those caring for PLWD as “courtesy stigma”, stemming from association with a stigmatised person, or as “self-stigma” following internalisation of social attitudes.

Scott (2022) demonstrates how disclosure decisions are mediated by an awareness of dementia-related stigma and the potential to be ascribed a stigmatised identity. As a consequence, diagnosis can be concealed. Stigma also influenced how PLWD perceived themselves, with both felt (Corrigan and Watson, 2007) and enacted (Scambler, 1989) stigma experienced from internalisation of public stigma and experiences of discrimination, respectively. Similarly, a systematic review by Herrmann et al. (2018) is suggestive of stigma holding significant consequences for PLWD and their carers, including deterring help-seeking, delayed diagnosis and treatment, social isolation, stress, low self-esteem, and caregivers fearing judgement and experiencing shame. Caregiver stress may result in coping difficulties and in turn negative emotions (and potentially behaviors) towards PLWD (Aboseif and Woo, 2020), further impacting PLWD’s experience of multiple facets of total pain.

For these reasons, reducing dementia-related stigma could improve access to care, support, and quality of life for PLWD and their carers. Despite an increasing amount of research highlighting its pervasivity, universality and negative consequences, research focusing on effective, evidence-based interventions to reduce dementia-related stigma is lacking (Kim et al., 2019; Herrmann et al., 2018).

#### 2.2.2 The sociality of dementia

Although stigma and social death are highlighted as features of living with dementia, there exist more hopeful accounts of the impact of dementia on social aspects of life. Phinney (2011:260) found that although PLWD interpreted their diagnosis as a “death knell” involving realisation that they were nearing the end of life, in the present they maintained involvement in activities that they found meaningful (“the things I did before”) and experienced their lives as essentially unchanged. Hennelly et al.’s (2021) review indicates that many PLWD find new roles (for example, informal supportive and caring roles in care settings), as a strategy allowing a sense of occupation and purpose, together with enjoying new social relationships (with formal caregivers and other residents in care settings, for example). Indeed Brannelly (2011:690) suggests that PWLD can be perceived as occupying an “emergent social identity”.

Connection (sense of belonging, safety), attachment and feeling accepted, valued and loved by others, are also identified as aspects of social relationships in the lived experience of wellbeing for PLWD, which can be used as resources to sustain positive functioning and equilibrium to underpin wellbeing (Clarke et al., 2020). Sawyer et al.’s (2019:7) work too presents social relationships as a resource. Drawing on evidence from their realist review and Bourdieu’s (1986) work on social capital, they argue that whilst a state of liminality follows diagnosis, social capital influences outcome in terms of whether this state is overcome, extended, or individuals live “in the shadow of the fourth age” (characterised by complete dependency). Social capital serves to socially orientate PLWD and facilitates the expression of a new identity. The authors suggest that cohesive support networks can advocate for the wishes of PLWD through knowledge exchange between community members, whose expertise (built through care provision), allows PLWD to maintain their sense of purpose and active citizenship. The review found that as dementia progresses however, social networks can dwindle, leading to a state of liminality resulting from uncertainty regarding the future, to which biomedical and public discourse depicting dementia as “death in the realm of the living” can also contribute (*ibid.*:5).

#### 2.2.3 The double jeopardy of dementia – dementia as an (anti)social disease

Social death and stigmatisation are social processes. Dementia precipitates these processes, however social death and stigma also contribute to the meaning and experience of dementia in a recursive fashion. Socially constructed impacts create a double jeopardy for PLWD, as a biomedical and social disease.

Language used to describe dementia plays a prominent role in shaping perceptions of it, and can contribute to social death (George, 2010). Stemming from the “language of warfare”, George (2010:586) argues that PLWD are viewed in accordance with primal metaphors, as “victims” “ravaged” by disease that “attacks” the brain. Similarly, the totalising parlance of “loss of self”, “living death” and dominant tropes of “burden” and “crisis” position PLWD in a way that removes them from human networks, engendering social death. George (2010) argues therefore that semantic choice is morally imbued. Similarly, Sweeting and Gilhooly (1997:99) suggest that social death is interpersonally and socially determined by whether PLWD are treated as a non-or liminal people prior to biological death. Returning to positioning theory (Sabat and Harré, 1992), it might be argued that perceiving and describing PLWD as socially dead may lead to treatment as such, risking a self-fulfilling prophecy.

#### 2.2.4 Implications of the social aspect of total pain for person-centred dementia care

In a similar vein to the psychological impact of dementia, the way in which the social impact of dementia is perceived and articulated has implications for the experience of social pain, its management, and person-centred care.

The preceding analysis suggests that the way that dementia is (negatively) socially constructed can inform (negative) social outcomes. Efforts to manage the social impact of dementia and reduce potential sources of social pain, should therefore focus on contributory social processes—changing dominant social tropes, narratives and discourses, for example. Professions, organisations and educational institutions should challenge the predominant dementia discourse of “death in the realm of the living” Sawyer et al. (2019:5). George (2010) suggests that reconsidering the language used in the context of dementia can transform thoughts, attitudes and behaviors, contributing to a more life-affirming and socially connective reality, rather than perpetuating a narrative complicit in fuelling fear, sadness and social death. They argue that language should orientate attention towards the humanity of PWLD and how they can be included in social activity. Fostering opportunities for PLWD to purposefully participate in community activities, for example, by facilitating interaction with children through reading, crafting, singing and sharing life history, as a means of establishing a role in networks that can preserve purpose, status and quality of life (*ibid.*). As PLWD (are permitted to) become more socially active, socio-political structures and dynamics influencing public perceptions of, and discourse surrounding dementia, may begin to change (Sawyer et al., 2019).

Similarly, if personhood is to be facilitated, healthcare professionals must see PLWD as socially alive actors, with each interaction an opportunity for enablement or disempowerment (Brannelly, 2011). Aboseif and Woo (2020:637, 638) for example, suggest that a “perceived lack of reciprocity” in PLWD contributes to stigma amongst healthcare professionals and the view that attempts to engage PWLD socially is a “wasted effort”. They suggest however that adapting expectations based on previous levels of social functioning allows for more “subtle displays of reciprocity” to be realised. Acceptance of change in social identity, allowing this to grow and fostering active citizenship, rather than attempting “to maintain a historical identity”, may allow a more positive experience of caregiving, acknowledging that health and illness co-exist, focusing less on loss and grief (Sawyer et al., 2019:10).

Once again, this analysis suggests that a binary approach to understanding the impact of dementia is insufficient for accommodating the complexity of and socially constructed nature of aspects of dementia realities. Acknowledgement of change (in social relationships, role, status, identity), and social construction, rather than “loss”, would appear critical to supporting PLWD towards active social participation and status, as part of person-centred care. With Vernooij-Dassen et al. (2018), we suggest that dementia presents both opportunities and limitations, existing in a dynamic balance. Since social aspects influence this balance, it becomes a social responsibility to ensure that this is weighted in favour of opportunities.

### 2.3 Synthesis and strategies (existential issues) and the spiritual domain of total pain

This section considers the meaning of total pain in the context of living and dying with dementia, in relation to spiritual pain, and the synthesis and strategies aspects of the 6S model. Synthesis and strategies are presented together in the 6S model and relate to existential issues that can arise during the dying process. Synthesis is retrospectively oriented, referring to an individual’s reflection on and summary of their life. Strategies are prospectively oriented and focus on remaining life. These processes include life story-telling, considering the meaning of life and what happens beyond death, and planning for the end of life.

Synthesis and strategies (existential issues) correspond with spiritual needs and the spiritual domain of total pain. Existential issues associated with dementia therefore form potential sources of spiritual pain. This section focuses on literature relating to existential issues in dementia, to explore the spiritual aspect of total pain in the context of living and dying with dementia, and implications for person-centred care.

#### 2.3.1 Existential threats and opportunities

In influencing the self and social relations, dementia can have a profound influence on one’s sense of purpose and meaning in life, as existential issues. Despite this, there is a relative paucity of research directly considering existential aspects of experience for PLWD (Mak, 2011; Dewitte et al., 2022; Phinney, 2011).

Meaning in life can be defined as “the personal experience that life is comprehensible and fits together (coherence), that life matters and is worth living (significance), and that one has valuable life goals and direction (sense of purpose)” (Dewitte et al., 2022:1155). The existential impact of dementia can been interpreted in a threatening way. Cheston et al. (2015:416, emphases added) for example, suggest that in understanding the impact of dementia, there is a “need to understand the way in which dementia acts as an existential *threat*. Dementia can *compromise* identity, *challenge* independence, prompt social *isolation* and *threaten* our ability to find meaning and purpose in life.” They found however that social connectedness, self-esteem and sense of meaning can mediate individual responses to this “threat”, concluding that those “who feel good about themselves” and “feel more connected to the world” are better able to see purpose and meaning in their life.

Others interpret dementia’s influence in terms of continuity of and new meaning. The “retained narratives” of participants in Phinney’s (2011:258) study demonstrate how despite living with mild to moderate dementia, life can be “meaningful in the same ways as before”, with continuity in themes that defined life prior to diagnosis. Participants understood “their lives to be unchanged in important and essential ways”, and continued meaningful engagement with the world. In addition, “shifting narratives” tell of new meanings developed. An instance is reported for example, where despite being aware from previous caregiving experience of the realities of advanced dementia, a participant reframed their situation in such a way that opened new possibilities of meaning. New narratives of the self were constructed, allowing them to continue life with equanimity. Phinney (2011:262) suggests that whilst PLWD may not be able to do all things that are important to them, they may still appreciate and enjoy life, through acknowledging a shifting orientation—that “different things will matter” and life will remain worth living. That is, PLWD “may no longer understand the bare fact of the palm tree, but will appreciate its beauty as a thing of nature”. Phinney (2011:262) suggests that through creativity and this “imaginative turn”, a new story, self-understanding, comfort and acceptance is found, engagement with the world is continued, and potential despair associated a future meaningless life is avoided, even when loss in relation to things currently considered to be important is experienced.

Similary, Wolverson et al.’s (2016:695) meta-synthesis suggests that the experience of dementia can foster growth and transcendence in relation to proffering opportunities for learning, self-understanding, and new contributions stemming from a dementia diagnosis (e.g., participation in research, sharing experience and knowledge with others). Active preservation of continuity associated with the self and identity can contribute to a re-evaluation of meaning and human growth. They suggest that some individuals are able to “transcend” and place “dementia in context”, minimising the impact that it has on their life. These sentiments are echoed by PLWD in Clarke et al.’s (2020:4) study. As part of making sense of their dementia in the context of the meaning of their life, participants experienced “shifting perspectives towards existential meanings”, including transcendence of their illness and spiritual growth. They felt content with life as it currently was and experienced positive emotions in the “here and now”.

The findings of Dewitte et al. (2022:1155, 1163) extend qualitative research and challenge assumptions of the requirement for intact cognitive abilities within prevailing conceptualisations of meaning. Their work suggests that “high levels of meaning can be experienced even when cognitive abilities are quite impaired”, supporting the idea “that meaning can arise at a pre-reflective level as an implicit bodily felt sense (Gendlin, 1962/1997; Greenberg and Pascual-Leone, 2001). This felt sense involves affective and cognitive processes, but does not require higher-order cognitive abilities to be intact.” Further, their finding of lower depressive symptoms reported at one year by those with “a higher presence of meaning”, demonstrates the importance of a sense of meaning in life to psychological wellbeing in PLWD.

#### 2.3.2 Implications of the spiritual aspect of total pain for person-centred dementia care

Several implications can be drawn from this analysis of existential issues in the context of dementia, for the management of spiritual aspects of pain (spiritual care) in PLWD.

Once again, the literature depicts a complex landscape in which rather than simply retaining or “losing” (in this case) meaning in life, PLWD report continuity of, shifting, and new meanings, together with spiritual growth, even in the context of more advanced dementia. References to the “here and now” (Clarke et al., 2020:4) and appreciation of beauty despite being unable to comprehend the thing to which it refers (Phinney, 2011), echo Dewitte et al.’s (2022) observations regarding meaning at the pre-reflective level, which is not dependent on higher-order cognitive abilities being intact. These accounts challenge the assumption that dementia erodes meaning in life and offer more impetus and scope for the development of “meaning interventions” (Dewitte et al., 2022:1155) that healthcare professionals can incorporate into person-centred practice, to support existential and spiritual wellbeing. Indeed, this suggests that such interventions *should* become part of routine person-centred care, as a moral imperative. Dewitte et al. (2022:1163, 1164) argue that “the possibility and importance of experiencing meaning in life” warrants further research into the potential of meaning-focused interventions for PLWD and the need to incorporate a felt, intuitive dimension to conceptualisations of meaning. On a day-to-day level, they suggest that interventions might include “activities that promote a sense of contributing something worthwhile” and reminiscence programs. Phinney (2011:266) offers that retained narratives might be harnessed by caregivers as an interpretive frame in the provision of spiritual care, to foster opportunities and direction “for more meaningful engagement” and support.

Improving existential wellbeing may also have a positive impact on other aspects of total pain and challenging behavior, symptomatic of unmet need. Sutin et al. (2022) found that a sense of purpose in life in PLWD offers a protective effect and was associated with fewer behavioral and psychological symptoms of dementia. Sense of purpose was associated with a lower risk of psychological symptoms including less periodic confusion, depression and uncontrolled temper. They conclude that focusing interventions on purpose in life may assist in promoting and maintaining wellbeing more broadly, reducing behavioral and psychological symptoms of dementia and improving quality of life overall for both PLWD and their carers.

### 2.4 Self-determination (autonomy) and the integration of total pain domains

This section considers the meaning of total pain in the context of living and dying with dementia, in relation to the self-determination component of the 6S model, relevant to all domains of total pain. The 6S model suggests that self-determination relates to an individual’s need to actively participate in their life, including at the end of life, through acknowledgement of beliefs and values. Where self-determination is not possible, it suggests that decision-making should become the responsibility of a proxy decision-maker, in accordance with patient preferences.

The dementia literature tends to discuss self-determination in relation to issues of autonomy, therefore this section focuses on autonomy in dementia care.^2^ In the 6S model, self-determination corresponds with psychological needs but also transects all aspects of total pain (see Figure 1). For the purposes of this analysis, reflecting the particular salience, complexity and unique nature of issues of self-determination for PLWD, we discuss self-determination broadly as an integrative concept, of relevance to all domains of total pain, with psychological pain instead having been discussed in section 2.1, in relation to the self (see Figure 3).

Due to its integrative nature, the challenges that dementia presents to self-determination can be interpreted as a potential source of all aspects of total pain. The following analysis draws on literature relating to dementia and autonomy to explore this cross-cutting aspect of the experience of total pain in the context of living and dying with dementia, and its implications for person-centred care.

#### 2.4.1 Individual and relational autonomy

Exercising autonomy is considered a key problem for PLWD, and its loss a greatly feared aspect of the disease (Wolfe et al., 2021; Phinney, 2011). The literature relating to the impact of dementia on autonomy broadly mirrors the nature of that identified in previous themes. Biomedically oriented analyses assume a progressive and inevitable loss of autonomy, focusing for example on the prediction and rate of its loss (Lechowski et al., 2005; Nicolas et al., 2022). Alternative literature approaches the issue through considering, in effect, how autonomy for PWLD is socially and relationally constructed, and in turn, how it can be supported. Rigeax (2011a, 2011b) and Davidson (2019) draw attention to two potential ways of conceptualising autonomy that illuminate this division, and the implications of each for PLWD. At the canonical pole, autonomy relates to individual competencies (memory, reflexivity, rationality), involving “rational decision-making by a stand alone self”, situating “others” as a potential threat (Rigeax, 2011a, 2011b:203). This rationalist definition dominates approaches to autonomy (including in healthcare) and ultimately excludes PLWD from its exercise (Wolfe et al., 2021; Rigeax, 2011a). The focus on independence within this traditional approach can also conflict with the inter-dependent nature of caring relationships experienced by PLWD (Wolfe et al., 2021).

Conversely, as Rigeax (2011a) explains, the relational pole considers the external conditions of autonomy. According to this perspective, whether PLWD have access to autonomy is dependent on the interpersonal relations, policies and institutions surrounding them, and the extent to which they afford the opportunity for its exercise. The focus of autonomy is therefore shifted from individual towards social preconditions. In this instance, the “other” is not threatening *a priori* and rather becomes a potential resource to support autonomy. This second conception allows for an ethic of care that maximises individual capabilities and affords PLWD access to autonomy for longer. In this way, a relational conception of autonomy is conducive to its exercise by PLWD (Davidson, 2019).

Rigeax (2011a) suggests that respecting autonomy canonically conceived of, involves proxy best-interest decision-making based on previous interests, following assessment of incompetence. The concern of relational autonomy however is a shared rather than individual one. Although individual autonomy is emphasised in law, relational autonomy emphasises mutual inter-dependence in decision-making and recognises that autonomy can be both constrained and enabled by social relationships (Davidson, 2019). Relational autonomy therefore allows for the possibility that autonomy in PLWD can be facilitated and enabled (Davidson, 2019) and becomes a question of practice rather than deliberation – of how to facilitate decision-making in an individualised way (Rigeax, 2011a).

Further, Wolfe et al.’s (2021:1885) research is suggestive of “a range of perspectives indicating a more complex relationship than a purely binary individual-relational distinction”. The facets PLWD considered to be important in the meaning of autonomy included independence (mirroring individual conceptualisations of autonomy), but also decision-making support from family and entrusting others with decisions-making, and adapting to limitations, reflecting an emphasis on autonomy as inclusion, supported in the context of trusted relationships. Others strongly rejected the notion of reliance on “other” (family or professional) however, seemingly associated with motivations of self-protection and anxiety.

#### 2.4.2 Implications of self-determination as a source of total pain, for person-centred dementia care

The way in which autonomy is conceptualised influences the impact that dementia is considered to have on it, the extent to which PLWD are considered to have access to it, and potentialities for facilitation by formal and informal caregivers. These things in turn have implications relevant to all facets of total pain.

The preclusion from exercising autonomy in its canonical form, but retention relationally where external conditions allow, serves as another example of how aspects of the impact of dementia, and of “loss” or “maintenance”, are socially constructed. A relational approach affords healthcare professionals the opportunity to facilitate autonomy, as part of person-centred pain management. Rigeax (2011a) suggests that this might be fostered through practises of explaining, listening, answering questions, and by developing and displaying sensitivity and reactivity to verbal and non-verbal indicators of preference.

van der Weide et al. (2023) found that successfully facilitating autonomy was dependent on the competency of healthcare professionals and an effective triadic relationship between them, PLWD and their relatives. Reassurance that PLWD have others to support and care for them may assist in allaying fears about declining autonomy (Phinney, 2011). In order to be effective however, relational autonomy must be recognised as legitimate by healthcare professionals, as must the dialogical nature of its construction, paternalism avoided, and institutions, policies and education must be supportive, including by providing required time and resources (Rigeax, 2011a, 2011b). In turn, reflecting the integrative nature of self-determination, the adoption of a relationally based narrative of autonomy may assist in addressing social pain (reducing stigma and isolation) and psychological pain (related to the self), associated with issues of autonomy.

Davidson (2019:1) cautions however that a relational approach may be more applicable during earlier stages of dementia, and at later stages, the inability “to make a legally autonomous decision (even with support) should be honestly acknowledged”. They suggest that such acknowledgement is supportive of autonomy, and care should then be aimed at respecting remaining and previous expressions of autonomy in decision-making processes, using a principle-based or best interests framework. It should also be recognised that a relational approach entails the risk of others’ views dominating and marginalising those of PLWD, particularly in later stages, where the approach risks not being “autonomous in any true sense”.

Also highlighting important caveats, Wolfe et al. (2021:1886, 1888) argue that the issue of (supporting) autonomy for PLWD should not be regarded as a “one-size-fits-all” phenomenon, and that autonomy cannot be considered simplistically in terms of a debate between “a purely independent or relational autonomy”. There is a need to critically reflect upon individual approaches to autonomy currently used as a basis for dementia care practice, and consider the social context, preferences, and the role and nature of relationships (present and historical) in the lives of PLWD, in considering how autonomy can best be supported.

## 3 Discussion

The preceding sections have explored the meaning of total pain in the context of living and dying with dementia, and its implications for the provision of person-centred care. Using a palliative care framework and extant literature we have critically considered how each aspect of total pain manifests and may be experienced in dementia realities.

Our analysis has demonstrated that the way in which we understand, articulate and approach the psycho-socio-spiritual impact of dementia (that is, on the self, social relations, existential issues and self-determination), has implications for the experience of psycho-socio-spiritual pain itself (total pain) and possibilities for its management. Recalling the interactive and inter-related nature of total pain (1.3), psycho-social-spiritual experience can in turn influence physical pain and vice versa.

### 3.1 Understanding beyond binaries and the “loss-deficit paradigm” —towards an ethic of integration and balance

Our analysis has highlighted the complexity and nuance of the experience of living and dying with dementia, which we argue cannot be accounted for by a dualistic “continuity or loss” (of self, social relations, meaning and autonomy) understanding. In highlighting a plethora of possibilities (e.g., for adaptation, maintenance, change, evolution, fragmentation, emergence, flux, concealment, preservation), and the socially contingent nature of dementia realities, we have challenged binary understandings and dominant deterministic discourses of “loss”, demonstrating that more subtle, discrete, hopeful (and less painful) outcomes are possible. Simplistic binary and “loss” understandings overlook this fruitful “middle ground” of possibilities, the role that caregivers (and society more broadly) play in mediating the impact of dementia on total pain, and with it, opportunities for changing outcomes and improving quality of care. Importantly, appreciation that aspects and degrees of both continuity and loss, together with a multitude of alternatives and socially constructed aspects, are experienced by PLWD creates new scope for supportive interventions, at individual and social levels.

We therefore advocate for an ethic of care within person-centred dementia pain management, that challenges the privilege of THE ‘LOSS-DEFICIT PARADIGM’ (WOLVERSON ET AL, 2016:696) - OF dominant biomedical “loss” understandings of disablement, decline and deficit, in favour of a more sensitive, functionally-oriented, balanced view, that carves a space for and prioritises the development of supportive care practices, and better quality of life for PLWD and their carers. Rather than focusing on (and looking for) “loss” as an inevitable outcome, greater attention should be paid to critically interrogating the process, mechanisms and outcomes of the “loss” model, and its adequacy for characterising the realities of living and dying with dementia. More nuanced (and optimistic) perspectives require incorporation in assessments of the impact of dementia, in order to avoid viewing PLWD in homogenous, reductive and stereotypic terms (Scott, 2022), and to negotiate more sophisticated, balanced understandings that better reflect the realities of dementia, and create opportunities for recognising and supporting personhood, and caring better within a palliative, person-centred framework. With its focus on promoting understanding and wellbeing, and valuing and maximising strengths, resources and functioning, this agenda is entirely consistent with a person-centred approach (Wolverson et al., 2016).

This agenda contributes to a growing body of work committed to challenging constraining understandings. Wolverson et al. (2016:676 emphasis added) argue that the prevalence of discourses of fear, stigma, negative media portrayals and pessimistic healthcare professional attitudes documented in the dementia literature, obfuscate more positive interpretations of dementia realities. They stress that strengths, capabilities and the potential for positive experiences and fulfilment “in spite of or even *because of*” living with dementia should be highlighted, as a means of de-stigmatising dementia and enhancing person-centred care, wellbeing and care quality. These sentiments are echoed by Grenier et al. (2017:318) who call for perspectives that address socio-cultural challenges to interpretations of dementia as “failed” and “frailed”, and suggest that modifying the constitution of subjects, and responses in care practices and social structures, can contribute to social change.

Phinney (2011) argues that if we are to improve our understanding of and better support dementia realities, we must look beyond either/or propositions including continuity/loss and intelligibility/incoherence, towards accounts of how PLWD live both possibilities, with caregivers acknowledging that PLWD can be who they are currently, in addition to who they are becoming. Indeed McParland et al. (2017) argue that movement away from a dichotomised approach should entail acceptance of the complex and diverse experiences associated with living with dementia, with respect, acceptance and inclusion central to this agenda. The dissolution of binaries can also be seen in the work of Grenier et al. (2017:144, 320 emphasis added), who argue that in order to approach vulnerability in a way that translates into the potential for a supportive response, there is a need to recognise that communication and autonomy (for example) “may look *different* in later life, and be as much socio-cultural as biological” – different from current interpretations, or differently enacted or communicated, rather than compromised. Here, the task for healthcare professionals is to value the experiences, convictions and lives of PLWD, even when what is being expressed appears “foreign” or “unknowable”.

### 3.2 “Tragedy” and “living well”

Deficit-oriented approaches to dementia (focusing on loss, decline, debility and death), have been collectively referred to as reflecting a “tragedy discourse”, which continues to exert considerable influence in healthcare practice (Dewitte et al., 2022; McParland et al., 2017:258; Grenier et al., 2017). Caution has been urged however in promoting “positive” discourses (e.g., “living well”, “active” and “successful” ageing) as an antidote to the nihilism of the “tragedy discourse”, since this can (paradoxically) serve a dichotomising function (Grenier et al., 2017; McParland et al., 2017:259, 260).

McParland et al. (2017) suggest that in response to the “tragedy discourse”, an alternative discourse of “living well” has become evident in policy internationally, emphasising independence (and the avoidance of dependence), cognitive and physical activity, autonomy, and the recognition and support of remaining strengths and enduring personhood. Whilst ostensibly offering a positive reframing, possibility, hope and the opportunity to change perceptions, McParland et al. (2017:262) and Grenier et al. (2017) problematise this narrative, arguing that it risks marginalising PLWD who may be assessed as “unsuccessful”, “failed” and “frailed” according to “living well” criteria. That is, PLWD may be considered to have “failed the living well test”, in turn legitimising social exclusion and different rules, systems, care locations and decision-making rights.

With McParland et al. (2017:266), we suggest that opposing “tragedy” and “living well” discourses essentially compete with one other on a discursive continuum, serving to falsely dichotomise dementia, and do not therefore (together or singularly) encourage a balanced discourse reflective of dementia realities. “Living well” and similar discourses simply prescribe “a new set of social expectations” that risk encouraging PLWD to continue to “fight” to meet social definitions of a “normal” life of “value” (e.g., in terms of the self, social relations, meaning and autonomy) rather than interrogating these definitions in light of living with dementia, in a way that acknowledges difference, change and adaptation. In this way, McParland et al. (2017:266) observe that “living well” discourses reflect society’s continued denial of the complex, multiple realities of PLWD and may perpetuate not only “divisive public perceptions” but “create division among people with dementia themselves”, as a consequence of differing abilities to meet “living well” criteria – in accordance with different locations on the dementia trajectory, levels of support, and intersecting social variables and identities (e.g., see Roes et al., 2022), for example.

In challenging discourses of “loss” (and tragedy), we do not therefore advocate for a simplistic antonymous and equally flawed “positive” substitution. Neither do we attempt to deny, minimise, invalidate or undermine the challenges encountered by PLWD and their carers (George, 2010) by means of promoting a “myopic positivity” (Levitt, 2023)—which too engenders marginalisation and the loss of opportunity for understanding and support. In reframing and reimagining, we are not denying but balancing. We intend neither to trivialise nor dramatise the impact of dementia (German Ethics Council, 2012), and emphasise the importance of looking for “presence” in addition to acknowledging “absence”, and being realistic about limitations, whilst not assuming that “they are more global than they are” (Mahon and Sorrell, 2008:114). Discourses of “loss” and “continuity”, “tragedy” and “living well”, are each considered compelling, with the denial of either one disingenuous, since all communicate an element of “truth” associated with dementia experience (McParland et al., 2017:266). We encourage recognition of pleasure *and* suffering, potential *and* limitation, vitality *and* vulnerability, and the supportive opportunities *and* challenges this creates in the realities of living and dying with dementia [McParland et al., 2017 (drawing on the work of Baars and Phillipson, 2014); Mahon and Sorrell, 2008].

### 3.3 The (in)adequacy of total pain for facilitating this agenda—development of a balanced model of total pain

Based on our analysis, we suggest that the concept of total pain presents both opportunities and challenges in terms of its utility for pain management within person-centred palliative dementia care.

#### 3.3.1 The adequacy of total pain—contributions

The concept of total pain is valuable *a priori* in providing the theoretical resources to support an orientation to care based on a more holistic understanding of dementia, that moves beyond its conceptualisation as a biomedical disease and the associated primacy of physical pain, towards viewing it as a multifaceted, bio-psycho-socio-spiritual phenomenon, entailing the potential for pain holistically and interactively conceived of. Total pain allows consideration of each holistic facet as valid and valuable in its own right – not as dependent or determined by its contribution to addressing physical pain, and the relative contribution of physical pain is not privileged. In this way, total pain emphasises the need to acknowledge, assess and address pain comprehensively, in turn offering the potential to more comprehensively meet the palliative needs of people living and dying with dementia. Being underpinned as it is by unmet need, a total pain approach may therefore present non-pharmacological solutions to reduce challenging behavior, fostering better quality of life for PLWD and their carers.

In driving this exploration of the impact of dementia on the self, social relations, existential issues and self-determination (as a means of considering what total pain means in the context of living and dying with dementia), the concept of total pain has facilitated the development of an ethic of care (described in 3.1 and 3.2) informed by insights around complexity, social contingency, nuance and the need for balance. In the process, “total pain” has fostered an appreciation of the critical context of total pain management in the context of dementia, and the realities of living and dying with dementia more broadly, together with the development of implications for practice that hold the potential to improve care (presented at the end of each pain domain analysis, and in 3.1, 3.2). This further supports the utility of total pain for improving dementia care quality.

#### 3.3.2 The (in)adequacy and paradox of total(ising) pain—a praxis critique

Whilst the concept of total pain has driven this exploration, more specifically, it is the critical nature of the analysis, challenging binary and “loss” understandings of the “pain” of dementia, that has yielded insights around the need for a more nuanced, balanced approach, creating space for the development of supportive care practices that hold the potential to improve quality of care. This criticality has been developed from synthesising evidence and insights from extant literature relating to the psycho-socio-spiritual impacts of dementia, as potential sources of total pain. We question the extent to which the adoption of a total pain approach (in its current form) in practice is able to foster an equivalent criticality that ultimately challenges binary and loss orientations in pain management—and therefore its capacity to facilitate the translation of critical insights into changes to pain management practice.

Our analysis has demonstrated the importance of language in the social construction of dementia realities. We argue that inherent within “pain” traditionally conceived of as it is, as an “unpleasant sensory and emotional experience” (International Association for the Study of Pain, 2020:no page), is a negative bias which remains *even when* pain is conceptualised holistically, as “total” pain. “*Total* pain” orientates towards different facets of pain, but “total *pain*” still emphasises *prima facie* a focus on “unpleasant” experiences, more readily aligned with negative affect and outcomes (for example, “distress”, “sadness”, “fear”, “worry”, “loss”, “lack”, “decline”, “deficit”), within each pain facet. We argue therefore that in the absence of the critical dementia context introduced in this paper (emphasising complexity, contingency, nuance and the need to look beyond loss towards balance), “total pain” risks orientating caregivers disproportionately towards “loss” and the deficit-oriented approach that has been challenged. This negative bias observed within the concept of “pain” is compounded by the negative bias in orthodox understandings of the realities of living and dying with dementia, which forms an interpretive context for pain management, increasing the likelihood of an unbalanced “pain” assessment, when adopting a “total pain” approach in practice.

Whilst total pain can be considered beneficial in proffering holistic, more comprehensive pain management, in the absence of the critical context central to understanding the meaning of total pain in the context of living and dying with dementia, it may not encourage the nuanced, balanced consideration of the impact of dementia in assessing pain that underpins the ethic of care advocated, and which allows for the development of supportive interventions and change needed to improve quality of care for PLWD and their carers. The application of total pain in dementia care practice, may risk perpetuating totalising understandings of dementia—as overwhelmingly bio-psycho-socio-spiritually painful in nature, shrinking the critical and supportive space that a nuanced and balanced understanding creates. Paradoxically, in turn, it may serve to entrench and perpetuate the pain that it is attempting to illuminate and address.

#### 3.3.3 Expanding the “total”—balancing total pain for use in dementia care through incorporation of a critical holistic approach

To improve utility, we suggest that the concept of total pain be situated within a critical context when introduced and applied to dementia practice – using a potted version of the critical narrative surrounding total pain management in the context of dementia care, together with its implications for practice. Further, our analysis suggests that a practice model of total pain in the context of living and dying with dementia should foster not only the assessment and management of pain holistically conceived of, but also incorporate the holistic assessment and management of each discrete aspect of total pain. That is, in addition to conceiving of pain holistically (bio-psycho-socio-spiritually), holistic, critical and balanced consideration is required *within* each facet of total pain itself. Consideration of the “pain” of living and dying with dementia relative to each domain requires a balanced approach attentive to both “painful” and more “functional” aspects of experience that capture strengths and capabilities, celebrate personhood and promote wellbeing – aspects that are fostering physical health, self-image, social relations, meaning in life, autonomy and proactively preventing bio-psycho-socio-spiritual pain and improving quality of life more broadly.

**Figure 4:**
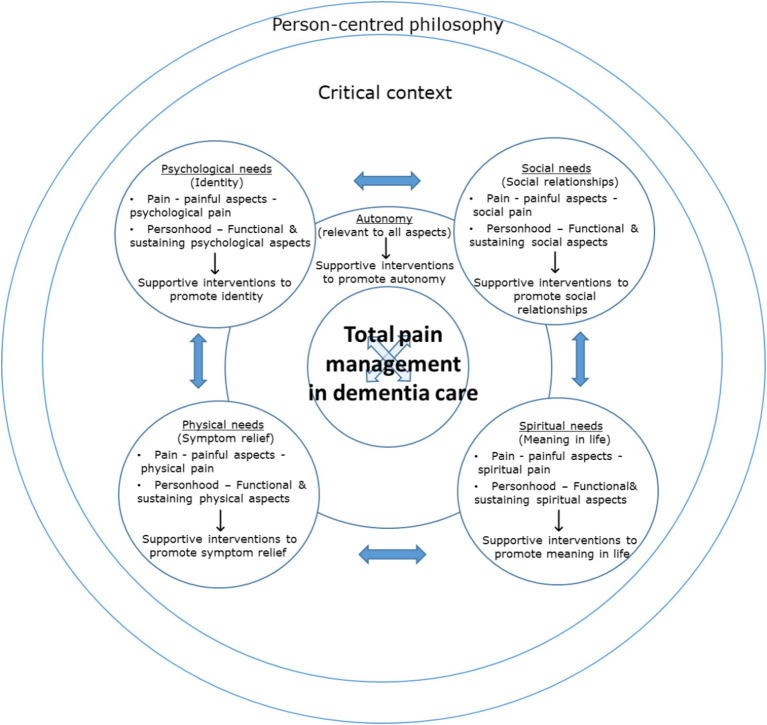
A balanced model of total pain in the context of living and dying with dementia, incorporating a critical, holistic approach (practice model). Developed from Figure 3.

**Figure 5:**
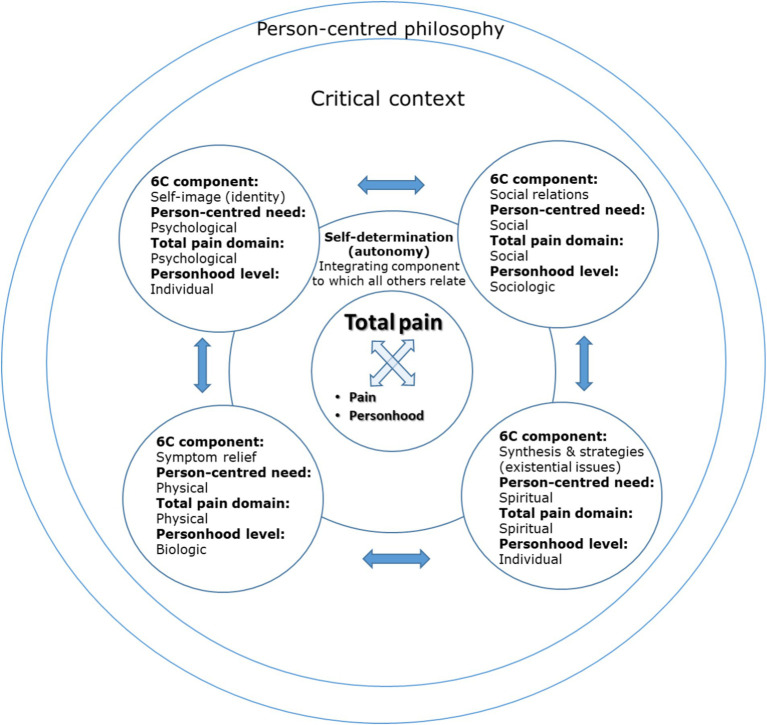
A balanced model of total pain in the context of living and dying with dementia, incorporating a critical, holistic approach (theoretical model). Developed from Figure 3.

Figure 4 depicts a balanced model of total pain for use in dementia pain management practice, incorporating a critical holistic approach, that could be used to facilitate application to practice and translation of positive outcomes. This practice model might be accompanied by the framework of addressing *presenting* pain, and *preventing* potential pain through *promoting* strengths and capabilities, and *celebrating* personhood (presenting, preventing, promoting and celebrating). Figure 5 depicts the balanced model in the theoretical context of the combined model of total pain.

We suggest therefore that (total) pain management in the context of living and dying with dementia necessitates looking beyond “pain” and even “total pain”, by including additional holistic and critical elements to support a balanced approach that ensures appreciation of personhood, and scope for the development and implementation of supportive interventions and change. The “total” in “total pain” should be understood as extending to incorporate a critical, holistic approach, not only to pain but to each facet of pain itself – “pain” is not only bio-psycho-socio-spiritual but functionally balanced, better reflecting the nature of “pain” in living and dying with dementia, and the critical context in which it is located. This is depicted in the models of Figures 4, 5 as a dual focus on “pain” and “personhood”, located within a critical context.

### 3.4 Future directions

#### 3.4.1 The need for further research and intervention development

There is a need to develop specific, accessible, practical, actionable and evidence-based supportive interventions and strategies, beyond pharmacological “solutions”, in each domain of total pain, to support a balanced approach to total pain management in dementia. There is also a need to consider how interventions might be implemented and evaluated, together with how they can be articulated and potentially modified to allow use across care settings (home, care homes, hospitals) by all staff (including unregistered workers) and informal caregivers. To facilitate this, more research is needed that explores complex bio-psycho-socio-spiritual realities of living and dying with dementia, from the perspectives of PLWD themselves (e.g., Phinney, 2011; McParland et al., 2017; Clarke et al., 2020). There is a need to explore the co-occurrence and nature of challenges and continuities (Wolverson et al., 2016), the nature of growth, gratitude, transcendence, self-compassion and wisdom *etcetera* in future research, in order to elucidate a “clearer understanding” of the experiences of PLWD and how they ‘might “flourish” in the “here-and-now”’ (Wolverson et al., 2016; Grenier et al., 2017:328; Clarke et al., 2020:10). Developing interventions for total pain management in dementia as a complex bio-psycho-socio-spiritual endeavour would correspondingly benefit from a multi-disciplinary, multi-professional and multi-agency approach.

The concept of cultural pain does not feature in the bio-psyco-socio-spiritual total pain model and has not therefore been explicitly explored in this paper. Further work might explore the meaning of cultural pain in dementia, together with implications for pain management practice. This is important given the increasingly multi-cultural context of patient populations and healthcare, and the imperative for cultural competence in care provision. Further, dementia is a global issue and holistic needs are universal, the exact nature and manifestation of total pain, and the critical contexts surrounding dementia, are socio-culturally influenced and will differ across international contexts. The principles outlined in this paper are therefore relevant internationally but further work would be needed to establish transferability of more specific aspects of the analysis.

Starting at undergraduate level, to facilitate change more broadly in line with the recommendations of this paper, the education of healthcare professionals should encompass teaching around nuance, complexity and the social contingency of the nature and meaning of pain in the context of living and dying with dementia, and its location in a critical context (the balanced model of total pain). This teaching is relevant to curriculum areas of (a) the psychosocial context of health, illness and healthcare, (b) spiritual care, (c) dementia, and holistic and person-centred care more broadly, (d) palliative care, and (e) pain and pain management. There is also a need to develop equivalent “lay” educational materials and resources for caregivers.

#### 3.4.2 Whose pain is it anyway? Total, shared (dyadic) and vicarious pain

The concept of total pain includes recognition and consideration of the impact of (and on) informal caregivers. Whilst the role of informal caregivers has been touched upon in this paper, there has not been scope to explore the caregiver perspective. To ensure best outcomes, it is suggested that a total pain approach to living and dying with dementia requires dual consideration of the needs of both the PLWD and their caregiver(s) (the meaning of total pain in the context of *caring* for an individual living and dying with dementia), and how these needs interact (e.g., Berk et al., 2018). This is especially important given the primacy of informal caregivers in the dementia context, who are often under-supported. In addition to highlighting unique needs, the notion and nature of shared (or dyadic) pain – which might be used to refer to aspects that come into being and exist dynamically at the interface *between* PLWD and their caregiver(s) in a way that biological pain does not, may also be useful, where pain may take on a new form and meaning to the sum of the individual parts of the PLWD and caregiver(s)’ pain. If a negative bias is pervasive and prevalent within the experience and context of caregiving [as is suggested by Sawyer et al. (2019) and McParland et al. (2017)], the critical insights of this paper might be extended to the caregiving context. Indeed caregivers may experience pain, perhaps fuelled by the assumptions of the tragedy discourse, based on what they anticipate. It could be explored whether this “empathic”, “vicarious” or “anticipatory” pain may match or even exceed the pain felt by PLWD in actuality, in relation to the same issue, especially in later stages of dementia when cognition and reflective awareness may be more impaired.

#### 3.4.3 The need for broader narrative interruption and reconfiguration

There is a moral need to challenge broader social assumptions that dementia equates *de facto* with poor quality of life (Mahon and Sorrell, 2008). Reflecting the socially contingent nature of total pain and the mechanism of the self-fulfilling prophecy, caregivers and society more broadly should acknowledge their role and be attentive to ways in which they are (unwittingly) complicit in influencing total pain in PLWD, and opportunities for its management, in what they see (understanding), say (language) and do (behavior and care practices). Biases and preconceptions must be acknowledged (Mahon and Sorrell, 2008) and social interventions developed that promote the critical consideration of portrayals of (including language used to describe) the realities of PLWD in popular media, including charity campaigns, by (as McParland et al., 2017:263 suggest), emphasising the “normalities” of living and dying with dementia, as a means of balancing “established notions of the ‘empty shell’ or ‘living dead’ and stereotypes based on the most vulnerable PLWD.

Critically analysing the current constructs, relations, practices and contexts in which dementia is located, and challenging aspects that reinforce overly simplistic (negative) assessments of dementia in favour of balance and shared responsibility, can act to reposition and relocate understandings of dementia to a place where both vulnerability and joy can co-exist and “new types of care relationships” fostered (Grenier et al., 2017:327). By re-considering understandings of dementia and the “structures and relations of care” within which it is located, new responses to PLWD can be created (*ibid.*:328).

## 4 Conclusion

This paper has explored the meaning of total pain in the context of living and dying with dementia, and its implications for pain management in the provision of person-centred dementia care. Using a palliative care framework and extant literature we have critically considered the bio-psycho-socio-spiritual impact of dementia, as a means of exploring how total pain might manifest and be experienced in this context.

We have highlighted the complexity, nuance and socially contingent nature of the impact of living and dying with dementia. In doing so, we have challenged binary understandings of “continuity or loss” (e.g., of identity, relationships, autonomy), and totalising “loss” discourses, demonstrating that more subtle, varied and hopeful outcomes are possible.

Our analysis has demonstrated that the way in which we understand, articulate and approach the psycho-socio-spiritual impact of dementia (that is, on the self, social relations, existential issues and self-determination), has implications for the experience of psycho-socio-spiritual pain itself (total pain) and possibilities for its management. A balanced understanding of the impact of dementia (acknowledging *both* continuity and loss, alternatives and socially constructed aspects), creates new possibilities for supportive care practices to improve pain management and quality of life. The deficit-orientation of “total *pain*” may however (paradoxically) risk perpetuation of the pain that it attempts to address.

When applied to dementia care, we therefore suggest that “*total* pain” should be located and introduced within a critical context, emphasising complexity, contingency and nuance. The holistic focus of “*total* pain” should also be extended to incorporate a balanced consideration of both “painful” and more “functional” aspects of experience, consistent with and celebrating personhood, and with prevention in addition to alleviation of total pain.

Reflecting these arguments, we have introduced a balanced model of total pain incorporating a dual focus on “pain” and “personhood” within a critical context, to facilitate translation to practice. Work is needed to further explore the complex bio-psycho-socio-spiritual realities of living and dying with dementia, together with the development of specific evidence-based supportive interventions corresponding with each domain of total pain, to support a balanced approach to total pain management and with it, the opportunity to understand, communicate, support, maintain, include and care better.

## Data Availability

The original contributions presented in the study are included in the article/supplementary material, further inquiries can be directed to the corresponding author/s.

## Footnotes

^1^Österlind and Henoch (2021:3, 6) state that model components represent an individual’s holistic dimensions, needs and ‘also refer to the concept of total pain (cf. Saunders, 1978).’ They do not expand on the relationship to total pain however, aside from (very briefly) in relation to symptom relief and physical pain (‘Symptom relief alleviates the physical dimension of total pain’). We have therefore mapped the facets of total pain and the 6S model in accordance with how we consider the components best align. We have modified the self-image and self-determination aspects of the combined model to reflect the specific manifestations and meaning of total pain in the dementia context. We associate self-image with psychological needs (previously a unifying component only in the 6S model) and self-determination is designated as an integrating component (previously integrative and associated with psychological needs). Changes are further explained in the relevant sections of 2.0.

^2^Self-determination and autonomy are closely related concepts. Curtice and Exworthy (2010:151) suggest that autonomy is ‘the principle of self-determination whereby a person is allowed to make free choices about what happens to them, that is, the freedom to act and the freedom to decide, based on clear, sufficient and relevant information and opportunities to participate in the decision-making.’ In healthcare, autonomy traditionally depends on the notion of self-determination (Wolfe et al., 2021).
